# Fat cells gobbling up norepinephrine?

**DOI:** 10.1371/journal.pbio.3000138

**Published:** 2019-02-07

**Authors:** Vitaly Ryu, Christoph Buettner

**Affiliations:** Diabetes, Obesity, and Metabolism Institute, Department of Medicine, Icahn School of Medicine at Mount Sinai, New York, New York, United States of America

## Abstract

The sympathetic nervous system (SNS) controls key aspects of adipose tissue (AT) function through the release of norepinephrine (NE) and *beta* adrenergic signaling. Sympathetic tone is determined by NE release but also by the rate of extracellular NE clearance that historically has been believed to occur solely through solute carrier family 6 member 2 (SLC6A2) expressed on sympathetic neurons. Song and colleagues show that adipocytes can also clear NE through organic cation transporter 3 (Oct3). This contributes to our understanding of how adrenergic signaling is controlled in AT and also emphasizes the need to develop better methods to assess adrenergic signaling in vivo.

## Abstract 2

This Primer explores the implications of a recent study showing that fat cells can clear norepinephrine via organic cation transporter 3, thereby affecting the adrenergic signalling that plays a key role in controlling adipose tissue function.

The key role of adipose tissue (AT) in human biology and metabolic health is dramatically illustrated when AT is absent, a condition termed lipoatrophy. Patients afflicted with lipoatrophy lack the adipokine leptin and suffer the many aspects of lipotoxicity, such as extreme insulin resistance, dyslipidemia, and deposition of lipids in ectopic organs, manifesting as enormous hepatic steatosis. To the contrary, the most common metabolic diseases of our time, obesity and type 2 diabetes, are characterized by an excess of AT mass but with impaired AT function, which plays a key role in the pathogenesis of these metabolic diseases. There are three pivotal functions of AT—first, lipid storage (to prevent lipotoxicity) and the mobilization of energy through lipid breakdown, a process called lipolysis; second, the production and release of adipokines such as leptin and adiponectin, key orchestrators of energy homeostasis acting on many organs, including the brain; and third, energy expenditure through thermogenesis. AT function is controlled by a complex interplay of humoral and neural factors. Though humoral factors, such as leptin, cytokines, resistin, adipsin, angiotensinogen, adiponectin, etc., have long been recognized as major controllers, the role of the sympathetic nervous system (SNS) in regulating AT function has only emerged as critical relatively recently (for review, see [1]; see Fig 1).

**Fig 1:**
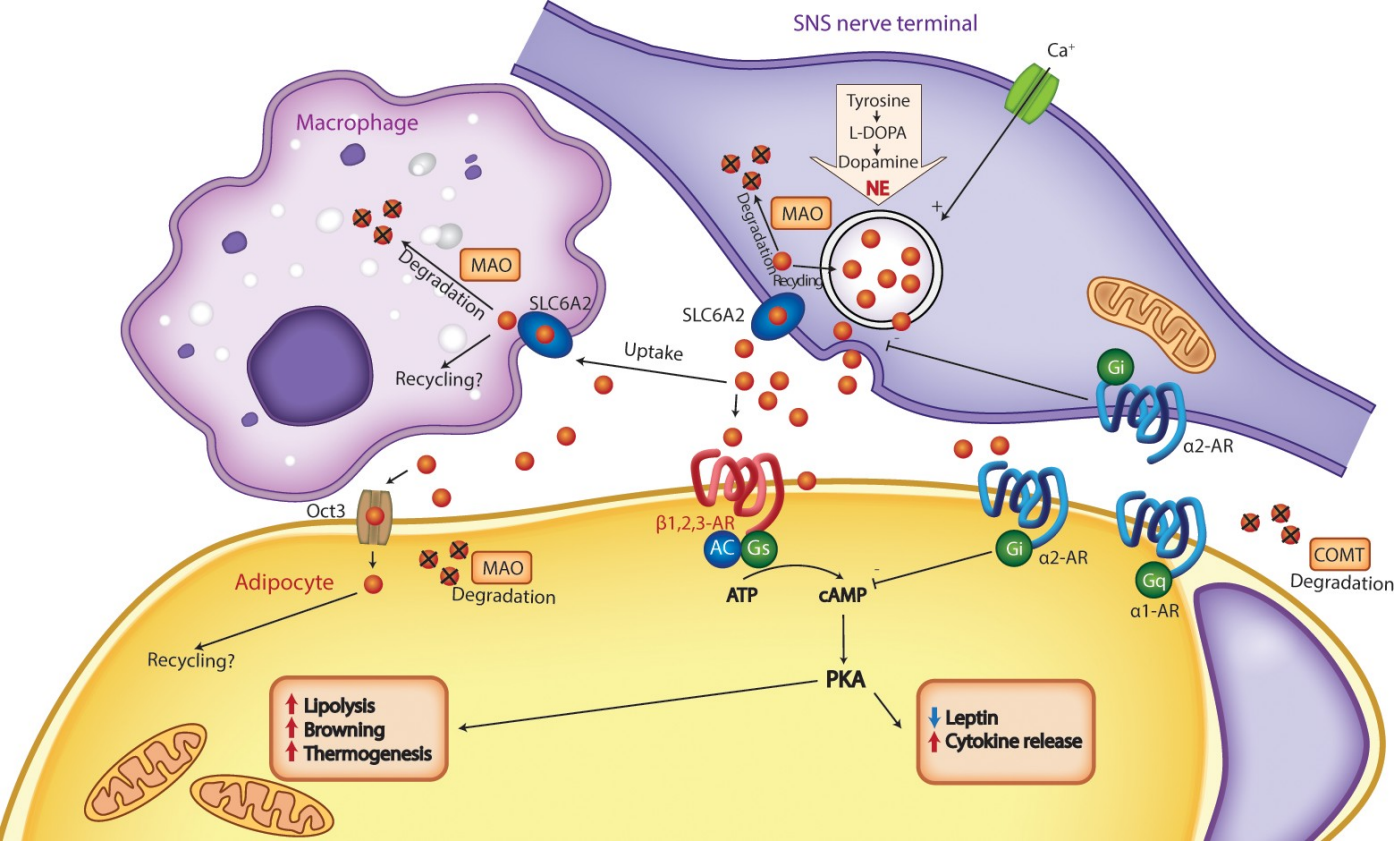
NE clearance in AT. NE released from the synaptic synapse can either signal through ARs or be cleared through uptake into either the sympathetic fiber or macrophages through SLC6A2 and then intracellularly either be recycled or degraded by MAO, or it can extracellularly be degraded through COMT. Reduced NE clearance increases AR signaling, in turn regulating key aspects of white AT function, such as lipolysis, thermogenesis, and release of adipokines. Song and colleagues show that Oct3 enables adipocytes to take up and degrade NE, which affects NE signaling. Ablation of Oct3 in adipocytes increases adrenergic signaling and thereby induces lipolysis, thermogenesis, and browning and reduces adiposity. AR, adrenoceptor; cAMP, cyclic adenosine monophosphate; COMT, catechol-*O*-methyltransferase; IL-6, interleukin-6; MAO, monoamine oxidase; NE, norepinephrine; Oct3, organic cation transporter 3; PKA, protein kinase A; SAM, sympathetic neuron-associated macrophage; SLC6A2, solute carrier family 6 member 2; SNS, sympathetic nervous system; TNF- *α*, tumor necrosis factor-*α*; AT, adipose tissue.

The principal transmitter/hormone of the SNS is the catecholamine norepinephrine (NE), synthesized from the amino acid tyrosine and stored in synaptic vesicles of SNS nerve fibers [2]. Upon sympathetic activation and depolarization-induced calcium influx, NE is released from SNS nerve terminals into the synaptic cleft, where it acts on the G-protein–coupled *alpha*- and *beta*-adrenoceptors (*α-* and *β-*ARs, respectively). In rodent AT, NE predominantly stimulates *β*3-ARs [3], activating adenylate cyclase, which increases cyclic adenosine monophosphate (cAMP), resulting in its accumulation and ultimately, leading to increased lipolysis, whereas in humans it appears that *β*2 and *β*3-ARs drive lipolysis [4,5]. Adipocytes also contain *α*1- and *α*2-ARs [6], which, upon activation, decrease cytosolic second messenger cAMP, which allows for a complex and differentiated regulation of cAMP signaling in AT that allows for depot and species specific regulation. Furthermore, *α*2-ARs expressed on the presynaptic sympathetic neuron inhibit the release of additional NE into the synapse and provide a negative feedback inhibition.

As mentioned above, the SNS controls key aspects of AT function. First, the SNS controls lipolysis chiefly through NE-induced *β*3-AR stimulation, which leads to the generation of the second messenger cAMP in the cytosol, which activates protein kinase A (PKA). In turn, the key lipolytic enzymes hormone-sensitive lipase (HSL) and adipose triglyceride lipase, which hydrolyze triglycerides into glycerol and free fatty acids, are activated through phosphorylation and recruited to the lipid droplet to induce lipolysis (for review, see [1]).

Second, the SNS controls the production and secretion of adipokines, such as leptin and adiponectin, and cytokines, such as interleukin-6 and tumor necrosis factor-*α* (TNF-*α*). For example, treatment with catecholamines in human adipocyte studies diminishes circulating leptin levels through signaling via *β*1*-* and *β*2*-*ARs [7] as does cold-exposure–induced SNS activation that results in a rapid decrease in AT leptin gene expression and plasma leptin levels [8]. Moreover, *β*3*-*AR stimulation drives white AT adiponectin exocytosis, which is impaired in obesity and/or type 2 diabetes possibly as a direct result of reduced *β*3*-*AR expression [9]. Similarly, *β*-AR stimulation has been reported to increase TNF-*α* [10] and interleukin-6 both in humans and in isolated human adipocytes. This emphasizes the role of the AT SNS in contributing to a spectrum of obesity-associated diseases via changes in adipokine production.

Third, the SNS induces browning of white AT through *β3*-AR stimulation [11,12], which refers to the conversion of white adipocytes to brown or beige adipocytes, characterized by increased mitochondrial density and increased uncoupling protein 1 expression, which uncouples oxidative phosphorylation from adenosine triphosphate synthesis, resulting in increased thermogenic capacity.

NE signaling is terminated by two mechanisms: NE can be degraded by catechol-*O*-methyltransferase (COMT) extraneuronally, i.e., outward of the synapse. The majority of NE (approximately 90%) is taken up by the presynaptic neuron through active transport via solute carrier family 6 member 2 (SLC6A2) monoamine transporter. After that cellular reuptake, NE can either be degraded by mitochondrial monoamine oxidase (MAO), or it can be sequestered in NE storage vesicles to be used again as a neurotransmitter; the latter then is described as NE recycling.

It follows that sympathetic tone is determined by two key parameters—first, NE release, a direct function of sympathetic activity, and second, through the clearance of NE, which is key to terminate adrenergic signaling. Classically, NE clearance was believed to occur exclusively via SLC6A2 expressed on sympathetic neurons [13]. But the sympathetic neuron is not the only cell type that expresses SLC6A2 in AT, nor is SLC6A2 the only transporter that takes up NE. Proinflammatory macrophages are unexpected carriers of SLC6A2 in AT and have recently been reported to take up and degrade NE through MAO [14]. Genetic deletion of SLC6A2 in macrophages increases AT browning and thermogenesis and reduces adiposity in mice, suggesting that proinflammatory macrophages can reduce NE signaling, contribute to sympathetic insufficiency in aging (which is associated with AT inflammation), and contribute to age-related reduction in adipocyte lipolysis [14,15].

Now, a report in the current edition of *PLOS Biology* shows that the organic cation transporter 3, Oct3 (Slc22a3) is highly expressed in AT in mice and enables cellular uptake of catecholamines in adipocytes with higher activity than previously estimated [22]. A previous study by Watts and colleagues described that perivascular AT exhibits NE uptake, which could be reduced by Oct3 inhibitors ex vivo [16]. Consistent with this observation, Song and colleagues find that Oct3 deletion in white adipocytes decreased NE uptake by 80%, but not in brown adipocytes, suggesting that Oct3 is required for NE uptake in white but not brown adipocytes. Importantly, after chemical sympathectomy, which destroys sympathetic fibers and obliterates sympathetic NE uptake, NE accumulation was higher in AT from Oct3 KO mice compared to WT mice, confirming a role of adipocyte Oct3 in NE clearing in AT. Adipocytes also express high levels of the NE-degrading enzymes Maoa, Maob, and COMT, indicating that NE clearance through adipocytes significantly contributes to NE clearance in AT. Oct3 ablation in AT enhances the effects of NE administration on increasing thermogenesis and lipolysis; Oct3 ablation also led to pronounced browning after prolonged cold challenge. The authors also identified reduced functional alleles of Oct3 in humans—variants that seem to be associated with increased basal metabolic rate—although it remains to be determined whether or not this is due to altered AT NE signaling, because the distribution of Oct3 expression according to GTEX differs from mice in which Oct3 expression is higher in the vasculature and nerves than in AT.

These are important studies because they change the way we think of NE signaling in AT. Historically, we thought of sympathetic tone, i.e., adrenergic signaling in AT, primarily to be a function of NE release and sympathetic NE uptake, but now we have to consider adipocyte in addition to macrophage NE uptake as an additional clearance mechanism. This raises the possibility, that by changing Oct3 activity, NE signaling could be altered and with it, AT function with potential therapeutic applications in obesity and other metabolic diseases.

These studies may also have implications for how we assess SNS activity in AT in vivo. NE turnover (NETO) is considered the gold standard because it provides a direct neurochemical measure of SNS activity within AT. This method utilizes the alpha-methyl-para-tyrosine as a competitive inhibitor of tyrosine hydroxylase (TH), such that without TH activity and thus NE synthesis, the endogenous NE levels in a particular tissue declines at a rate proportional to NE release [17]. NETO rests on the assumption that NE reuptake and storage occur only in SNS nerve terminals and that the decline of that activity is principally dependent on the release of NE from SNS terminals, thus providing an estimate of sympathetic NE release and sympathetic activity [18]. This assumption seems justified because only SNS nerve terminals seem to have NE storage vesicles [19]. However, there are examples of NE release from macrophages, and given that macrophages seem not to be able to synthesize NE de novo [20], this may represent recycling of NE. Similarly, there is a report that adipocytes can release NE [21], although the catecholamine measurements in this study are done by radioimmunoassay and not by the more reliable mass spectrometry-based method [21], so these findings need to be substantiated. If this can indeed be confirmed, then it would be justified to consider Oct3 part of an adipocyte NE recycling pathway. Furthermore, if indeed significant amounts of NE can be stored and released by both macrophage and adipocytes in addition to nerve terminals, it would reduce the validity of NETO as a direct estimate of SNS activity measure. But even if the ability of adipocytes and macrophages to store and release NE turns out to be insignificant—and thus NETO maintains its validity as an estimate of sympathetic NE release and sympathetic activity—the current study provides important evidence that sympathetic activity and NE release is only one determinant of NE signaling but that NE clearance, including via nonneuronal cells, is another important contributor to NE signaling. The latter is not assessed by NETO, and because NE signaling can be modulated by macrophage and adipocyte NE uptake and degradation, it is critical to evaluate adrenergic signaling in the adipocyte or any other target cell of the SNS such as hepatocytes or myocytes.

Therefore, this highlights the need to develop better methods to assess adrenergic signaling in vivo given the important role of adrenergic signaling in AT function and that sympathetic nerve activity is only one aspect of adrenergic signaling as the study by Song and colleagues elegantly shows [22].

## Abbreviations

AR: adrenoceptor
AT: adipose tissue
cAMP: cyclic adenosine monophosphate
COMT: catechol-*O*-methyltransferase
HSL: hormone-sensitive lipase
MAO: mitochondrial monoamine oxidase
NE: norepinephrine
NETO: NE turnover
Oct3: organic cation transporter 3
PKA: protein kinase A
SAM: sympathetic neuron-associated macrophage
SLC6A2: solute carrier family 6 member 2
SNS: sympathetic nervous system
TH: tyrosine hydroxylase
TNF- *α*: tumor necrosis factor-*α*
